# Peroxisome proliferator-activated receptor γ coactivator 1α maintains NAD^+^ bioavailability protecting against steatohepatitis

**DOI:** 10.1093/lifemedi/lnac031

**Published:** 2022-08-17

**Authors:** Weiyan Shen, Xingyong Wan, Jiahui Hou, Zhu Liu, Genxiang Mao, Xiaogang Xu, Chaohui Yu, Xudong Zhu, Zhenyu Ju

**Affiliations:** Key Laboratory of Aging and Cancer Biology of Zhejiang Province, Department of Pathology and Pathophysiology, School of Basic Medical Sciences, Hangzhou Normal University, Hangzhou 311121, China; Key Laboratory of Regenerative Medicine of Ministry of Education, Guangzhou Regenerative Medicine and Health Guangdong Laboratory, Institute of Aging and Regenerative Medicine, Jinan University, Guangzhou 510632, China; Department of Gastroenterology, the First Affiliated Hospital, School of Medicine, Zhejiang University, Hangzhou 310003, China; Key Laboratory of Aging and Cancer Biology of Zhejiang Province, Department of Pathology and Pathophysiology, School of Basic Medical Sciences, Hangzhou Normal University, Hangzhou 311121, China; Key Laboratory of Aging and Cancer Biology of Zhejiang Province, Department of Pathology and Pathophysiology, School of Basic Medical Sciences, Hangzhou Normal University, Hangzhou 311121, China; Zhejiang Provincial Key Lab of Geriatrics & Geriatrics Research Institute of Zhejiang Province, Department of Geriatrics, Zhejiang Hospital, Hangzhou 310013, China; Zhejiang Provincial Key Lab of Geriatrics & Geriatrics Research Institute of Zhejiang Province, Department of Geriatrics, Zhejiang Hospital, Hangzhou 310013, China; Department of Gastroenterology, the First Affiliated Hospital, School of Medicine, Zhejiang University, Hangzhou 310003, China; Key Laboratory of Aging and Cancer Biology of Zhejiang Province, Department of Pathology and Pathophysiology, School of Basic Medical Sciences, Hangzhou Normal University, Hangzhou 311121, China; Key Laboratory of Regenerative Medicine of Ministry of Education, Guangzhou Regenerative Medicine and Health Guangdong Laboratory, Institute of Aging and Regenerative Medicine, Jinan University, Guangzhou 510632, China

**Keywords:** nonalcoholic steatohepatitis, PGC-1α, SIRT2, NAD^+^

## Abstract

Hepatic metabolic derangements are pivotal incidences in the occurrence of hepatic steatosis, inflammation, and fibrosis. Peroxisome proliferator-activated receptor-γ, coactivator-1α (PGC-1α), a master regulator that mediates adipose metabolism and mitochondrial biogenesis, its role in hepatic steatosis and progression to steatohepatitis remains elusive. By surveying genomic data on nonalcoholic steatohepatitis (NASH) patients available in the Gene Expression Omnibus, we found that PGC-1α was significantly down-regulated compared with healthy controls, implicating the restoration of PGC-1α may ameliorate the hepatopathy. Using a hepatocyte-specific PGC-1α overexpression (LivPGC1α) mouse model, we demonstrated that PGC-1α attenuated hepatic steatosis induced by methionine–choline-deficient diet (MCD). Biochemical measurements and histological examination indicated less inflammatory infiltration, collagen deposition, NF-kB activation, and less lipid accumulation in LivPGC1α liver fed MCD. Further analyses indicated that the NAD^+^-dependent deacetylase sirtuin 2 (SIRT2) interacted with and deacetylated PGC-1α. Congruently, ablation of SIRT2 accelerated the NASH progression in mice fed MCD, while NAD^+^ repletion via its precursor mimicked the beneficial effect of PGC-1α overexpression and was sufficient to alleviate NASH in mice. These findings indicate that hepatic-specific overexpression of PGC-1α exerts a beneficial role in the regulation of steatohepatitis and that pharmacological activation of the SIRT2-PGC-1α-NAD^+^ axis may help to treat NASH.

## Introduction

Nonalcoholic fatty liver disease (NAFLD) is a rapidly growing epidemic with increased morbidity and mortality [1]. NAFLD encompasses a continuum, ranging from simple steatosis to nonalcoholic steatohepatitis (NASH) [2]. NASH is characterized by hepatocyte lipid accumulation accompanied by inflammation and varying degrees of fibrosis, which may eventuate in hepatocellular carcinoma [1]. Notably, NASH is the second most common indication for liver transplantation in the USA [3]. However, the pathogenesis of NASH development is still poorly understood.

Peroxisome proliferator-activated receptor-γ coactivator-1α (PGC-1α) is a transcriptional coactivator, which plays a key role in metabolic diseases by regulating the expression of genes related to fatty acid oxidation, oxidative phosphorylation, and mitochondrial function [4, 5]. Our previous work confirms that PGC-1α protects against hepatic steatosis and insulin resistance through IL-10-mediated anti-inflammatory response [6]. Congruently, inhibition of PGC-1α promotes hepatic inflammation in mice fed a high-fat diet by activating the IκBα-NFκB pathway [7], although other studies reveal that PGC-1α regulates the insulin signaling pathway and contributes to the development of NAFLD via increasing inflammation and oxidative damage [8, 9]. Therefore, the role of PGC-1α in progress from steatosis to steatohepatitis remains elusive.

The sirtuin family has been reported to regulate hepatic steatosis and NASH, although some findings are controversial [10–14]. Sirtuin 2 (SIRT2) is highly enriched in metabolically active tissue, including the heart, liver, and brain. Previous studies indicate that SIRT2 drives immunometabolic homeostasis linking its nicotinamide adenine dinucleotide (NAD^+^)-dependent deacetylase activity to many substrates [15]. Down-regulation of Sirt2 reduces the expression of β-oxidation and mitochondrial genes, with PGC-1α a potential target [16]. Interestingly, pharmacological inhibition of SIRT2 blocks the therapeutic action of NAD^+^ against NAFLD pathologies [17], while SIRT2 deficiency reprograms T-cell metabolism by targeting metabolic enzyme hyperacetylation, leading to a more effective antitumor immune response in the tumor microenvironment [18]. Given that the pathophysiological consequences of SIRT2 are highly contextual, the complexity of the role of SIRT2 in NASH requires further clarification.

NAD^+^ is a common coenzyme in all living cells [19]. It serves as a critical coenzyme for redox balance and a co-substrate for certain enzymes such as the sirtuins [19, 20]. Beneficial effects of NAD^+^ repletion on mitochondrial homeostasis, organismal metabolism, have been established [20]. Increased NAD^+^ levels protect organisms from metabolic diseases, including fatty liver, obesity, and aging [19, 21]. The supplementation of NAD^+^ precursor nicotinamide riboside reduces hepatocyte fat accumulation and attenuates liver fibrosis [22]. Inhibiting hepatic NAD^+^ content results in mitochondrial dysfunction and thus promotes alcoholic and NASH [23, 24]. One recent study indicates that PGC-1α induces *de novo* NAD^+^ biosynthesis and prevents acute ischemia/reperfusion-induced renal injury in mice [25], linking the PGC-1α–NAD^+^ axis to tissue homeostasis and stress response. Indeed, many studies have reported that PGC-1α is involved in the pathway of NAD^+^ biosynthesis and consumption in metabolic tissue such as muscle, liver, and adipose tissue. For instance, PGC-1α has been suggested to promote quinolinic acid synthesis via blocking α-amino-β-carboxymuconate-ε-semialdehyde decarboxylase, or to promote *de novo* synthesis via kynurenine aminotransferase activation. Moreover, PGC-1α may influence sirtuins, parps, and many enzymes in the NAD salvage pathway [26–29].

To investigate the role of PGC-1α in steatohepatitis and the underlying mechanism, we use the established hepatocyte-specific PGC-1α overexpression mice model [6] and demonstrate that PGC-1α is protective against hepatic impairment in mice fed MCD, probably involving an SIRT2-NAD^+^-dependent mechanism. These results indicate that the SIRT2-PGC-1α-NAD^+^ axis may serve as a therapeutic target in maintaining the liver metabolic function for appropriate adaptation to physiologic and pathological stress.

## Results

### Hepatic PGC-1α was reduced in NASH patients

**Figure 1.**
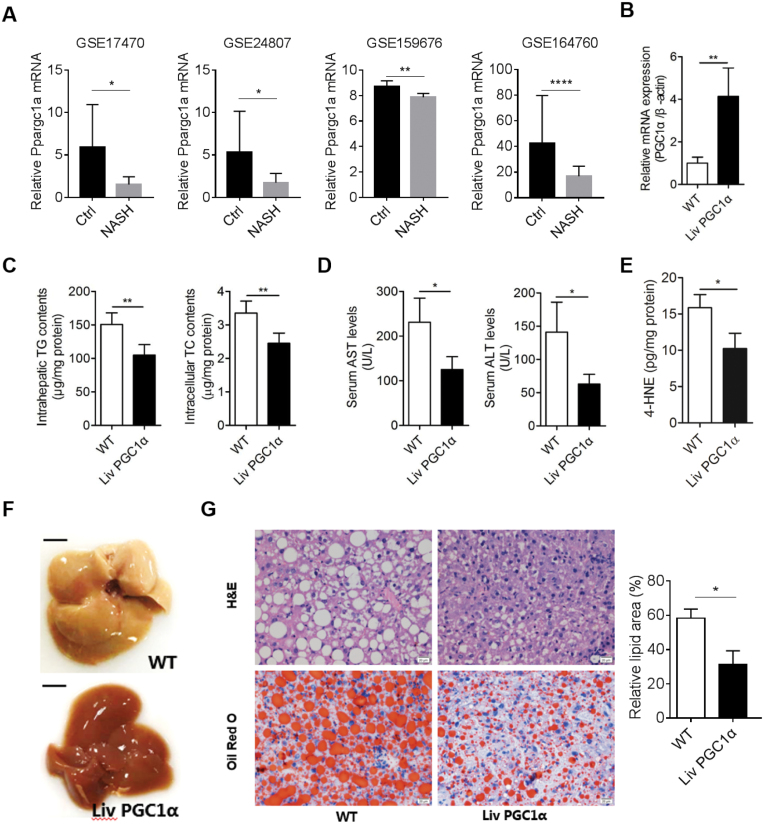
PGC-1α alleviates hepatic injury caused by MCD. (A) Expression profilings by array show the relative hepatic PGC-1α mRNA expression in healthy controls and NASH patients. Data were expressed as mean ± SD. *n* = 4–7 for GSE17470, *n* = 5 or 12 for GSE24807, *n* = 6 or 7 for GSE159676, *n* = 6 or 74 for GSE164760. (B) Relative PGC-1α mRNA expression of WT and LivPGC1α mice fed MCD for 5 weeks. (C) Intrahepatic TG and TC contents in LivPGC1α mice compared to WT mice fed MCD. (D) Serum AST and ALT levels in LivPGC1α mice compared to WT mice fed MCD. (E) 4-HNE quantification in LivPGC1α mice compared to WT mice fed MCD. (F) Representative morphology of WT and LivPGC1α mice fed MCD for 5 weeks. Bar = 500 μm. (G) Representative H&E, and Oil Red O staining from liver sections of WT and LivPGC1α mice fed MCD. Relative lipid area was calculated in LivPGC1α mice compared to WT mice fed MCD. *n* = 3–6 per group, except Fig. 1A. All data were expressed as mean ± SD. *P < 0.05, **P < 0.01, ***P < 0.0001.

To investigate whether PGC-1α participates in NASH progress, we first analyzed the public-available microarray data derived from four independent studies of liver specimens of healthy controls and NASH patients (https://www.ncbi.nlm.nih.gov/geo/query/acc.cgi?acc=GSE17470, https://www.ncbi.nlm.nih.gov/geo/query/acc.cgi?acc=GSE24807, https://www.ncbi.nlm.nih.gov/geo/query/acc.cgi?acc=GSE159676, and https://www.ncbi.nlm.nih.gov/geo/query/acc.cgi?acc=GSE164760). These studies validate that the PGC-1α transcriptional levels are significantly reduced than healthy controls (Fig. 1A), as per the analyses provided in GEO2R in the Gene Expression Omnibus (GEO) database, suggesting that boosting the PGC-1α expression is potentially feasible to intervene NASH.

### Hepatocyte-specific PGC-1α knock-in ameliorates steatohepatitis

MCD diet is widely used for the nutritional model of steatohepatitis that induces a severe form of steatohepatitis, including steatosis, hepatic inflammation, hepatocyte necrosis, and fibrosis. To assess the role of PGC-1α on the progression of steatohepatitis, a methionine–choline-deficient diet (MCD) was applied to the previously established mouse model of hepatic-specific PGC-1α overexpression (hereafter LivPGC1α) and littermates (hereafter WT) for 5 weeks. A 3-fold elevation of PGC-1α mRNA was seen in the liver of LivPGC1α mice compared with WT mice (Fig. 1B). This up-regulation of PGC-1α attenuated MCD-induced hepatic injury, as exemplified by lowered intrahepatic triglyceride (TG) and total cholesterol (TC) contents (Fig. 1C), as well as reduced serum levels of aspartate transaminase (AST) and alanine aminotransferase (ALT) vs that of WT mice (Fig. 1D). Notably, the liver of LivPGC1α mice exhibited a significantly reduced oxidative stress as measured by 4-hydroxy-2-nonenal (4-HNE) production compared with WT mice (Fig. 1E). In agreement, the development of a pale white liver color, which occurs with increased lipid content or steatosis, was attenuated in LivPGC1α mice and was corroborated by significantly hindered hepatic fat accumulation seen in the histological examinations (Fig. 1F and 1G).

**Figure 2.**
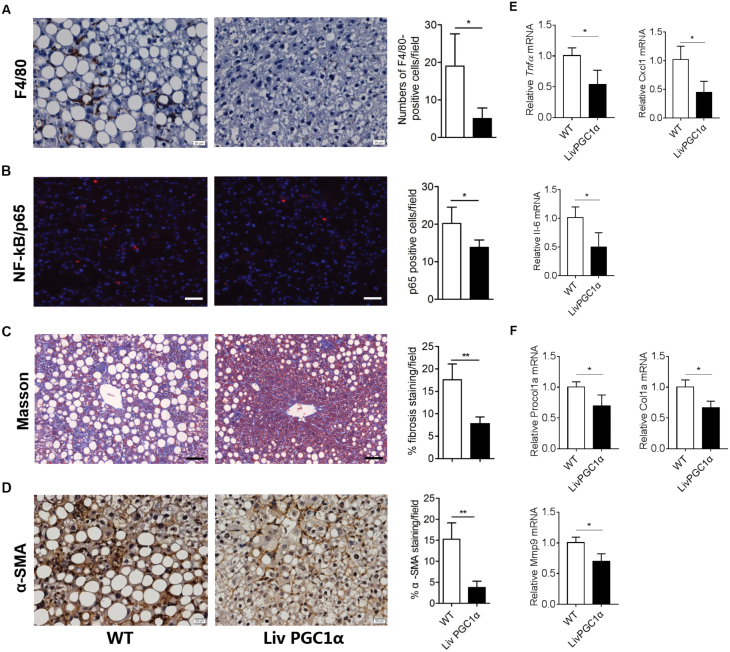
PGC-1α alleviates hepatic inflammation and fibrosis caused by MCD. (A) Representative images and positive counts of F4/80 (a pan marker of murine macrophages) immunohistochemistry in the livers of WT and LivPGC1α mice fed MCD (400× magnification). F4/80 positive cells were counted in randomly selected six areas for each section. (B) Representative images and positive counts of nuclear p65 subunit (a marker of NF-kB activation) translocation in WT and LivPGC1α livers (200× magnification). Scale bar = 20 μm. p65-positive cells were counted in randomly selected six areas for each section. (C) Representative images and positive counts of Masson’s trichrome staining in WT and LivPGC1α livers. Scale bar = 20 μm. Fibrotic areas were calculated in randomly selected six areas for each section. (D) Representative images and positive staining percentage of α-SMA immunohistochemistry in WT and LivPGC1α livers (400× magnification). α-SMA staining per field was quantified in randomly selected six areas for each section. (E and F) Relative proinflammatory (E) and fibrosis-related (F) gene expression of WT and LivPGC1α mice fed MCD. *n* = 4 per group. All data were expressed as mean ± SD. *P < 0.05, **P < 0.01.

To further verify whether PGC-1α relieves steatohepatitis induced by MCD, we performed immunohistological analyses in WT and LivPGC1α livers. Immunostaining of macrophage marker F4/80 showed a significant reduction of F4/80 positive cells in liver sections of LivPGC1α mice compared with those in WT mice (Fig. 2A). NF-κB subunit p65 nuclear translocation assay further validated less inflammatory activation in hepatocytes of LivPGC1α mice compared with that of WT mice (Fig. 2B). Moreover, LivPGC1α liver exhibited less collagen composition and hepatic fibrosis than those in WT mice as assessed by immunostaining of α-SMA and Masson’s trichrome, respectively (Fig. 2C and D). In line with this, proinflammatory genes (Tnfα, Il-6, and Cxcl1) and fibrosis-relate genes (Procol1a, col1a, and Mmp9) were all down-regulated in LivPGC1α livers compared with WT group (Fig. 2E and F). Together, these data indicate that PGC-1α could alleviate hepatic inflammation and fibrosis caused by MCD.

### PGC-1α knock-in attenuates mitochondrial dysfunction

**Figure 3.**
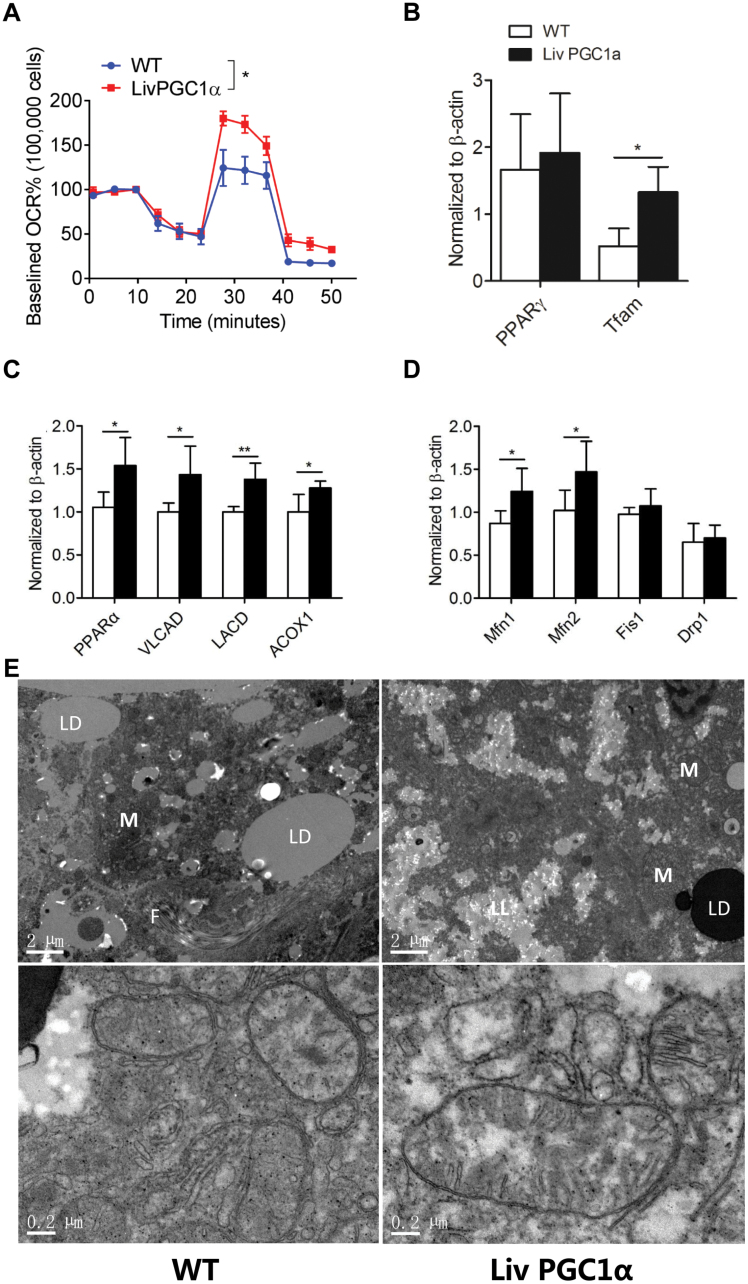
PGC-1α improves mitochondrial function in mice fed MCD. (A) Mitochondrial respiration measured in isolated primary hepatocytes from WT and LivPGC1α mice fed MCD for 5 weeks. OCRs were normalized to a same baseline (as 100%) per 100,000 cells. Two-way ANOVA was performed to compare the two groups. (B) Relative hepatic PPARγ and Tfam mRNA expression in WT and LivPGC1α mice fed MCD. (C) Lipid metabolism-related gene mRNA levels were measured in WT and LivPGC1α livers by quantitative real-time PCR. β-actin was used to normalize data. (D) Mitochondrial dynamics-related gene mRNA levels were measured in WT and LivPGC1α livers by quantitative real-time PCR. β-actin was used to normalize data. (E) Representative hepatic electronic microscopy images of WT and LivPGC1α mice fed MCD. Magnification: 2500× or 20,500×. F, fibrotic tissue; LD, lipid droplet; LL, lysed lipid; M, mitochondria. Comparison of WT and transgenic mice was performed using the Student *t*-test (*n* = 3–6 per group), except Fig. 3A. All results are presented as mean ± SD. *P < 0.05.

To address whether the beneficial effect seen above is mitochondrial-dependent, we next performed mitochondrial stress test by Seahorse. Indeed, LivPGC1α mice showed improved mitochondrial respiration in freshly isolated hepatocytes using a Seahorse XFe96 analyzer (Fig. 3A). In addition, although enforced expression of PGC-1α did not alter the expression of PPARγ, LivPGC1α liver exhibited a significantly increased expression of Tfam transcript level (Fig. 3B), PPARα and its target genes (Fig. 3C), and mitochondrial fusion-related genes (Fig. 3D) compared with the WT liver, suggesting an improved mitochondrial function and accelerated fatty acid oxidation upon PGC-1α induction. Consistent with these findings, electronic microscopy analyses demonstrated accumulated lipid droplets and fibrosis in the WT liver upon receiving MCD, while the LivPGC1α liver exhibited attenuated liver injury and relatively larger mitochondrial size (Fig. 3E).

### Down-regulation of PGC-1α is associated with reduced SIRT2 expression

**Figure 4.**
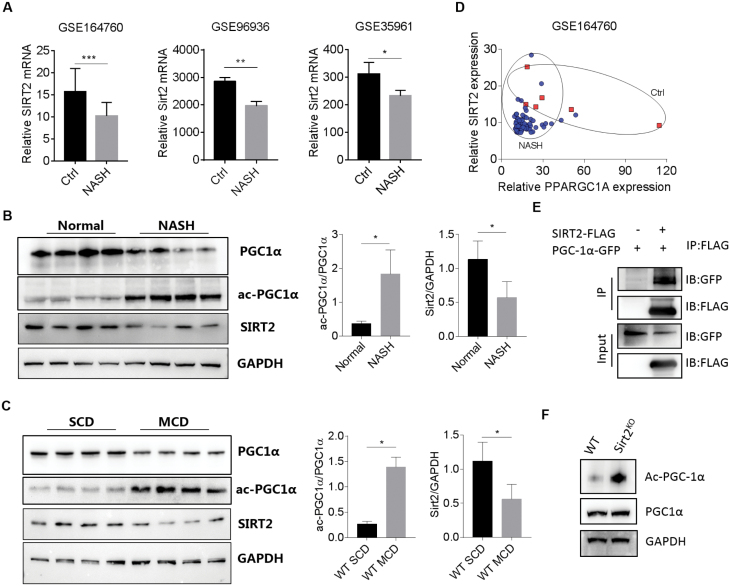
MCD-induced PGC-1α down-regulation is associated with reduced SIRT2 expression. (A) Expression profilings by array show the relative hepatic SIRT2 mRNA expression in human and mice. Data were expressed as mean ± SD. *n* = 6 or 74 for GSE164760, *n* = 3 for each group in GSE96936, *n* = 4 for each group in GSE35961. (B) Relative hepatic SIRT2, PGC-1α, and acetylated PGC-1α protein expression in healthy controls and NASH patients. Data were expressed as mean ± SD, *n* = 4 per group. (C) Relative hepatic SIRT2, PGC-1α, and acetylated PGC-1α protein expression in WT mice upon SCD or MCD treatment. Data were expressed as mean ± SD, *n* = 4 per group. (D) Reduced PGC-1α expression correlates with down-regulated SIRT2 expression in human NASH biopsies (data from GSE164760). *n* = 6 (Ctrl, red square) or 74 (NASH, blue dot). (E) Co-IP experiments of HEK293T cells cotransfected with Flag-SIRT2 and GFP-PGC-1α, using an anti-Flag antibody for IP, and anti-Flag and anti-GFP antibody for immunoblotting. (F) Co-IP experiments of WT and SIRT2 knock-out livers, using an anti-acetyl-lysine antibody for IP and PGC-1α for immunoblotting. All results are presented as mean ± SD. *P < 0.05, **P < 0.01, ***P < 0.001.

Acetylation and deacetylation modified by sirtuins have been suggested to play a role in hepatic diseases [30]. By surveying the expression of sirtuins in the GEO databases (https://www.ncbi.nlm.nih.gov/geo/query/acc.cgi?acc=GSE164760, https://www.ncbi.nlm.nih.gov/geo/query/acc.cgi?acc=GSE96936, and https://www.ncbi.nlm.nih.gov/geo/query/acc.cgi?acc=GSE35961), we identified a conserved down-regulation of SIRT2 in both NASH patients and mouse models (Fig. 4A). We also analyzed the relative mRNA transcript abundance of other sirtuin family members, i.e. Sirt1, Sirt3-7, in the GEO dataset (GSE164760). As is shown in Fig. S1, except Sirt7, we found all other sirtuins did not correlate with Ppargc1a expression in human NASH biopsies. Congruently, only Sirt2 and Sirt7 exhibited a significant down-regulation in NASH hepatic specimens versus that of healthy controls. In addition, HepG2 cells transfected with siRNA against Sirt1/2 exhibited significant lowered Sirt1 and Sirt2 mRNA levels in comparison to scrambled siRNA-transfected control, the total PGC-1α protein levels showed marginal changes; however, the ac-PGC-1α level increased upon Sirt1/2 knockdown (Fig. S2). Protein quantification in the liver specimens of humans and mice with steatohepatitis further confirmed markedly reduced SIRT2 expression, concurrent with increased acetylated PGC-1α-to-total PGC-1α protein levels (Fig. 4B and C). Importantly, we found a positive correlation between the hepatic expression of PGC-1α and SIRT2 in human NASH patients, where NASH patients exhibited synergistically lowered PGC-1α and SIRT2 expression compared to healthy controls (Fig. 4D). This prompts us to examine whether SIRT2 can interact and deacetylate PGC-1α. Indeed, immunoprecipitation manifested the interaction between SIRT2 and PGC-1α in HEK293 cells cotransfected with Flag-tagged SIRT2 and GFP-tagged PGC-1α (Fig. 4E). Next, we measured the abundance of acetylated PGC-1α in the livers of WT and SIRT2 knock-out mice. As expected, an up-regulation of acetylated PGC-1α protein expression was observed in SIRT2 knock-out liver homogenate, although the total PGC-1α level remains unchanged (Fig. 4F).

### SIRT2 knock-out accelerates MCD-induced hepatic injury

**Figure 5.**
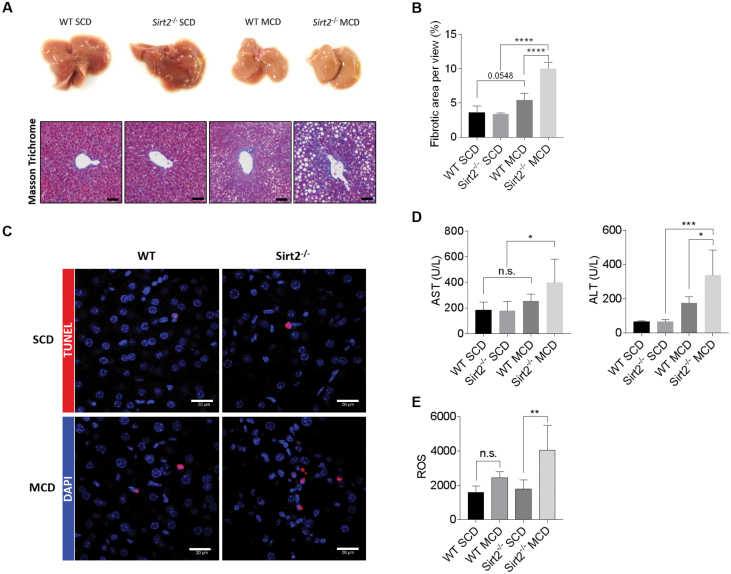
SIRT2 deficiency accelerates NASH phenotypes. (A) Representative morphology and Masson’s trichrome stainings of WT and SIRT2 knock-out mice fed SCD or MCD for three weeks. Scale bar = 20 μm. (B) Fibrotic area calculation in WT and SIRT2 knock-out mice fed SCD or MCD. *n* = 4 for each. (C) Representative images and positive counts of terminal deoxynucleotidyl transferase (TdT)-mediated dUTP nick end labeling (TUNEL) (a marker of apoptosis) in WT and SIRT2 knock-out livers fed 3-week SCD or MCD (400× magnification). Bar = 20 μm. (D) Serum AST and ALT levels in WT and SIRT2 knock-out mice fed 3-week SCD or MCD. (E) Relative intensity of reactive oxygen species (ROS) detected in isolated liver cells from WT and SIRT2 knock-out mice fed 3-week SCD or MCD. Comparison of WT and SIRT2 knock-out mice was performed using One-way ANOVA (*n* = 4–5 per group). All results are presented as mean ± SD. n.s. not significant, *P < 0.05, **P < 0.01, ***P < 0.001.

To investigate the pathophysiological role of SIRT2 in the progression of NASH, we subjected WT and SIRT2 knock-out mice to the MCD diet. SIRT2 knock-out led to an accelerated hepatic injury with the manifestation of NASH phenotype as early as 3-week MCD induction. Masson’s trichrome staining revealed a more severe lipid and collagenous fiber accumulation in SIRT2 knock-out liver compared to WT mice (Fig. 5A and B), concurrent with significantly increased hepatocyte death (Fig. 5C), and elevated serum AST and ALT levels (Fig. 5D). Furthermore, flow cytometry analysis of the isolated hepatocytes from SIRT2 knock-out mice exhibited a significantly increased ROS intensity than WT mice (Fig. 5E), suggesting more oxidative stress occurred in the SIRT2 knock-out livers.

### NAD^+^ repletion mimics the hepatoprotective effect of PGC-1α overexpression

**Figure 6.**
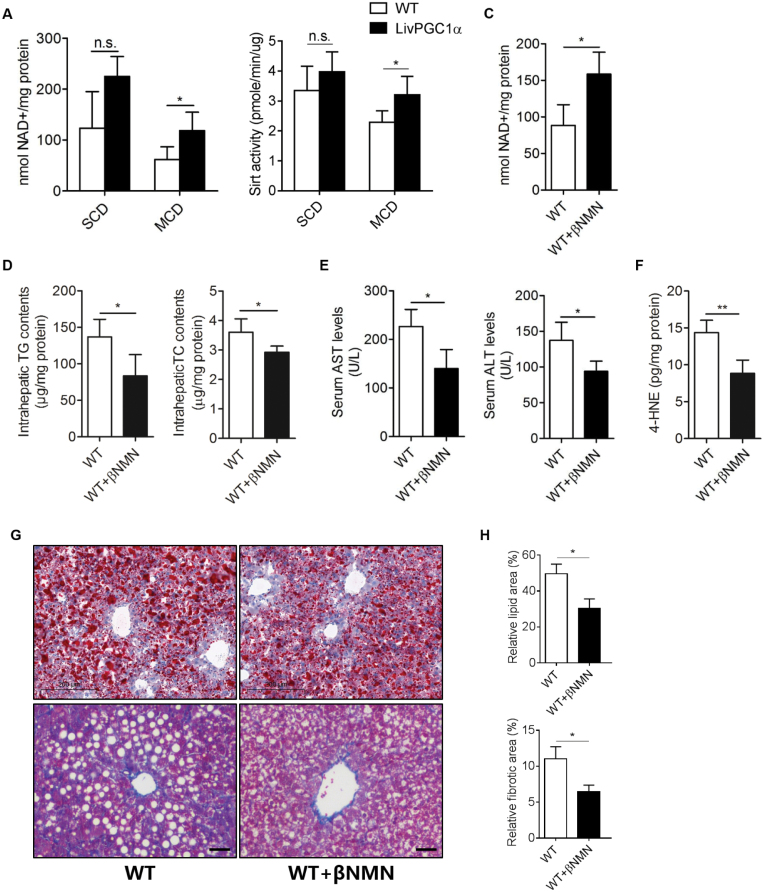
NAD^+^ repletion mimics the hepatoprotective effect of PGC-1α overexpression. (A) Hepatic NAD^+^ levels upon 5-week SCD or MCD treatment in WT and LivPGC1α mice. (B) Hepatic sirtuin activity in WT and LivPGC1α mice fed 5-week SCD or MCD. (C) Hepatic NAD^+^ levels in MCD-fed WT livers supplemented with or without 100 mg/kg/day of βNMN. (D) Intrahepatic TG and TC content in MCD-fed WT mice supplemented with or without 100 mg/kg/day of βNMN. (E) Serum AST and ALT levels in MCD-fed WT livers supplemented with or without 100 mg/kg/day of βNMN. (F) Measurement of hepatic 4-HNE production in MCD-fed WT mice supplemented with or without 100 mg/kg/day of βNMN. (G) Representative Oil Red O staining and Masson’s trichrome stainings of WT mice supplemented with or without 100 mg/kg/day of βNMN. Scale bar = 200 μm (upper) or 20 μm (bottom). *n* = 3–6 per group. All data were presented as mean ± SD. n.s. not significant, *P < 0.05, **P< 0.01 (H).

Since depletion of cellular NAD^+^ leads to the impairment of mitochondrial function [31], and PGC-1α is found to drive *de novo* NAD^+^ biosynthesis [25], we next hypothesized that PGC-1α knock-in might ameliorate NAD^+^ consumption. Indeed, 5-week MCD lowered the sirtuin activity and NAD^+^ level in the WT liver, both of which were alleviated by hepatic PGC-1α knock-in (Fig. 6A and B), suggesting that PGC-1α exerted a hepatic-protective effect partially via restoring hepatic NAD^+^ level induced by MCD. Notably, 3-month NAD^+^ repletion via its precursor β-nicotinamide mononucleotide (β-NMN) restored the hepatic NAD^+^ level (Fig. 6C). In addition, we observed significantly lowered intrahepatic TG and TC levels (Fig. 6D), and reduced serum AST and ALT levels in mice fed MCD (Fig. 6E). Furthermore, the β-NMN treatment group showed a significantly attenuated liver 4-HNE level than the control group (Fig. 6F). In line with these findings, the β-NMN group exhibited less lipid accumulation and fibrosis compared with that of WT group (Fig. 6G and 6H). These data suggest that β-NMN repletion mimics the protective role of PGC-1α overexpression in MCD-induced hepatic injury.

## Discussion

The pathogenesis for the development and progression of NASH is complex and has not been fully delineated. In this study, we identified that the SIRT2-PGC-1α-NAD^+^ axis might play a crucial role in steatohepatitis. We observed enhanced fatty acid oxidation and mitochondrial performance and subsequently reduced liver injury biomarkers in mice of hepatic-specific PGC-1α overexpression upon MCD treatment. Notably, such a beneficial effect of PGC-1α induction could be replicated by restoration of NAD^+^ level via its precursor repletion.

PGC-1α, a key co-factor mediating mitochondrial biogenesis and quality control, has been suggested to play an important role in the development of NAFLD. Lipid accumulation promoted TXNIP expression and PRMT1 activity, thereby inducing PGC1a level and adipogenesis-related gene expression, while excessive lipid accumulation directly activated TXNIP-NFκB signaling pathway, thus leading to inflammatory gene expression [32]. In line with this, another study documented that PRMT1 was found to induce PGC-1α mRNA expression through the recruitment of HNF-4α to the promoter region of PGC-1α, thereby attenuating hepatic steatosis via enhancing PGC-1α-mediated fatty acid oxidation [33]. In liver fibroblasts of NASH patients, lipid overaccumulation inhibits PGC-1α expression by activating endoplasmic reticulum stress protein CHOP [34]. Moreover, free fatty acid-induced lipid peroxidation stress and its associated oxidative stress, cell death, inflammation, and fibrosis could be attenuated by astaxanthin through an up-regulated FGF21 and PGC-1α expression in damaged hepatocytes [35]. However, whether PGC-1α takes part in the development of NASH is still indeterminate. Previous studies mainly focused on the effect of PGC-1α on metabolism-related phenotypes. Recently, PGC-1α was found to act as an important mitigator of apoptosis in TNFα or lipopolysaccharide-induced inflammatory environment [36]. Moreover, reduced hepatic PGC-1α increased oxidative damage and potentiated the transition from steatosis to steatohepatitis [9]. By generating mice with hepatic-specific overexpressed PGC-1α, we show here that in comparison to that of WT mice, a mild PGC-1α elevation in the liver is sufficient to ameliorate nonalcoholic steatohepatitis phenotype of hepatic inflammation, fibrosis, and oxidative stress by enhanced mitochondrial function and fatty acid oxidation in mice fed with MCD. Given that there are no approved drugs to treat NASH to date, PGC-1α represents a promising therapeutic target for translational studies in the future.

Mitochondria are highly dynamic organelles and their proper function is crucial for the maintenance of cellular homeostasis [37]. Hepatic mitochondria are structurally and molecularly altered in NAFLD [38]. Previous research showed that mitochondrial free cholesterol loading causes mitochondrial GSH depletion and sensitizes to TNF- and Fas-mediated steatohepatitis [39]. It is believed, at least in part, that mtDNA level relatively reflects mitochondrial number/density and therefore mitochondrial function [40]. We observed a slight increase in hepatic Tfam transcriptional level upon LivPGC1α induction, which may attribute to improved mitochondrial function and lipid homeostasis. We propose that MCD-induced mice increase hepatic mitochondrial stress and inflammation, therefore inhibiting mtDNA replication. Indeed, several lines of evidence demonstrate that increased oxidative stress down-regulates mitochondrial replicative genes and compromises mitochondrial biogenesis and function [41, 42, 43]. We also observed an improvement in mitochondrial function-related genes. Further investigation on this should be of great interest. Nevertheless, we find that LivPGC1α mice favor a better metabolic efficiency per mitochondrion, exerting improved fatty acid oxidation and fewer pathological indicators witnessed.

SIRT2 was known to regulate oxidative stress and energy metabolism via deacetylating many substrates, such as the transcription factor forkhead box O 3a (FOXO3a) [44] and the glucokinase regulatory protein [45]. It is also known to mediate inflammatory response via deacetylating NF-κB [46]. Interestingly, one recent study identifies that hepatocyte nuclear factor 4α can be deacetylated by SIRT2 [14]. Liver-specific SIRT2 deletion exacerbated metabolic dysfunctions in HFD-fed mice [14]. In line with this, we identified that SIRT2 ablation also deteriorates MCD-induced steatohepatitis, where SIRT2 may serve as an upstream of PGC-1α. However, another study suggested that SIRT2 inhibition suppressed carbon tetrachloride- or thioacetamide-induced hepatic fibrosis [10]. These conflicting results could be due to the different interventions to the animal models (i.e. profibrotic agents vs MCD diet), and the different cell types focused (i.e. hepatic stellate cells vs hepatocytes). Further investigations should pay attention to the comprehensive roles of SIRT2 in cell- and context-specific manners.

NAD^+^ is an important co-substrate for several enzymes, including the sirtuin family of NAD^+^-dependent protein deacetylases, PARP protein family, and the cyclic ADP-ribose synthases [20, 47, 48]. Meanwhile, NAD^+^ is consumed by these enzymes. Beneficial effects of increased NAD^+^ levels and sirtuin activation in the regulation of mitochondrial homeostasis have been established [49, 50]. Recent studies reported that PARP inhibition [23] and NAD^+^ repletion could reverse fatty liver disease in mice [22]. Other evidence also suggests a PGC-1α-dependent regulatory axis in directing *de novo* NAD^+^ biosynthesis under the context of acute kidney injury [25]. Therefore, we hypothesized that PGC-1α-mediated improvement in mitochondrial function and lipid metabolism in MCD-fed mice is partially due to restoration of NAD^+^ levels, which would boost antioxidant capacity and enhance metabolic performance in the impaired liver. In supporting this notion, we discovered a positive correlation between PGC-1α expression and NAD^+^ level in mice livers. In addition, β-NMN administration to mice fed with MCD exhibited attenuated liver injury matching with a restored NAD^+^ level. Given that supplementation of NAD^+^ or its precursors remains costly, the biosynthesis of highly efficient NAD^+^ boosters is of great interest.

**Figure 7.**
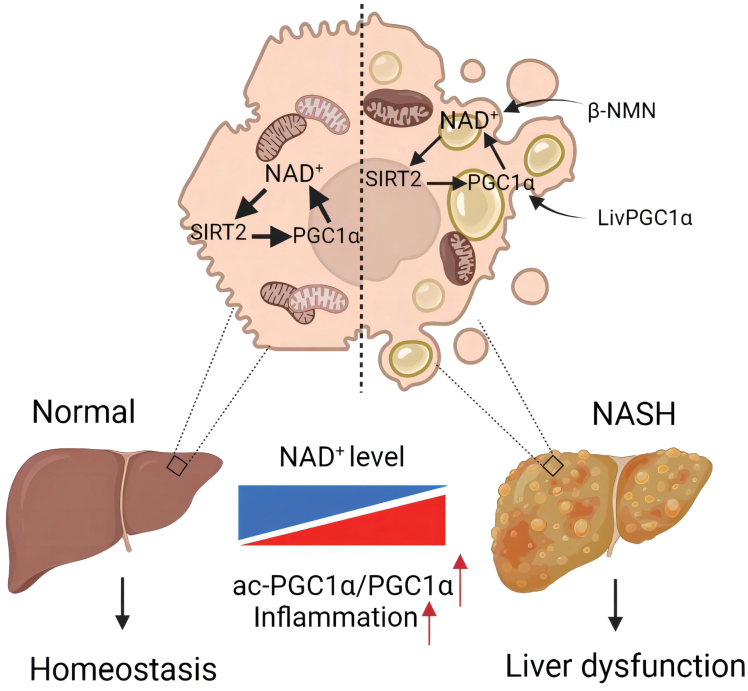
Graphical summary of the current study.

In summary, our data demonstrate that PGC-1α ameliorates hepatic steatosis and hinders progression to steatohepatitis, possibly via the SIRT2-PGC-1α-NAD^+^ feedback loop (Fig. 7). Specifically, PGC-1α augmentation confers an enhanced stress resistance manifested as increased antioxidant capacity and accelerated lipid metabolism in mice fed MCD. Thus, it should be considered to associate PGC-1α stimulation with inhibition of excessive NAD^+^ consumption as a potential strategy for counteracting NASH and other metabolic diseases.

## Research limitations

There are some limitations of the study. First, the classical dietary model of NASH, MCD diet, is used in the current study. Long-term feeding of MCD diet causes significant body weight loss and shrinked liver size proportionally, therefore cannot totally mimic human NASH phenotypes. Nevertheless, we treated mice in a limited timeframe (3–5 weeks) to minimize the side effects. Second, we focus on SIRT2 in the current study due to its higher mRNA and protein abundance in mouse liver in comparison to SIRT1. However, whether SIRT1 functions synergistically with SIRT2 in NASH requires further clarification. In addition, the sirtuin activity assay we performed did not distinguish SIRT1 or SIRT2 specifically. Third, a lack of SIRT2 conditional mouse model (i.e. hepatocyte-specific Sirt2^fl/fl^-Albumin Cre) may neglect the nonliver effect of SIRT2 KO in a paracrine manner.

## Materials and methods

### Animal experiments

Mice were maintained in a temperature- and light-controlled room with free access to food and water. LivPGC1α mice (LivPGC1α: PGC-1α^flox/+^ -Albumin^Cre^) and the control mice (WT: PGC-1α^flox/+^) were generated and maintained as described previously [6]. Male mice aged 12–16 weeks were fed either an MCD or a standard chow diet (SCD) for the indicated period, followed by humane sacrifice and the liver tissue and serum were collected for further analysis. All animal care and procedures were approved by the Animal Care and Use Committee of Hangzhou Normal University.

### Reagents

β-NMN was purchased from Bontac-Bio Inc. Customized primers were synthesized by Invitrogen. All other chemicals were purchased from Sigma-Aldrich unless elsewise stated.

### Histopathological analysis

Liver tissues were fixed in 10% neutral formaldehyde, dehydrated through an ethanol and xylene series, embedded in paraffin, sectioned, and then stained with hematoxylin-eosin (H&E). Hepatic steatosis was detected by Oil Red O staining in frozen liver sections. Liver fibrosis was evaluated by Masson staining in liver sections. Immunohistochemistry staining for F4/80 (ab6640; Abcam) and α-SMA (A5228; Sigma) was performed in formalin-fixed, paraffin-embedded liver sections according to the manufacturer’s instructions. Immunofluorescence staining for NF-kB (SN368; Beyotime) was performed in the frozen liver section according to the manufacturer’s instructions, and nuclei were stained with DAPI (Invitrogen).

### Quantitative real-time reverse transcription-PCR

Total RNA was extracted from liver tissues using TRIzol (15596026, Invitrogen). RNA was reversed transcribed to cDNA using PrimeScript™ RT Master Mix (RR036, Takara). Real-time PCRs were performed using the ABI 7900 machine (Applied Biosystems, Foster City, CA) and One Step PrimeScript™ RT-PCR kit (RR055, Takara). The relative gene expression shown in Table 1 was done in triplicate and calculated via the delta-delta Ct method (2^−ΔΔCT^ method). The primers used are listed in Table 1.

**Table 1.** Primers used for quantitative real-time PCR

Primer	Forward	Reverse
**β-actin**	GAAGATCAAGATCATTGCTCCT	TGGAAGGTGGACAGTGAG
**PGC-1α**	TATGGAGTGACATAGAGTGTGCT	GTCGCTACACCACTTCAATCC
**PPAR-α**	AACATCGAGTGTCGAATATGTGG	CCGAATAGTTCGCCGAAAGAA
**LCAD**	TTTCCTCGGAGCATGACATTTT	GCCAGCTTTTTCCCAGACCT
**ACOX1**	CCGCCACCTTCAATCCAGAG	CAAGTTCTCGATTTCTCGACGG
**VLCAD**	ACTACTGTGCTTCAGGGACAA	GCAAAGGACTTCGATTCTGCC
**PPAR-γ**	TGGCCACCTCTTTGCTCTGCTC	AGGCCGAGAAGGAGAAGCTCTTG
**Tfam**	GGAATGTGGAGCGTGCTAAA	GGTAGCTGTTCTGTGGAAAATCG
**Mfn1**	GGCGTGATTTGGAAAACAGTG	ATCATCTGCAGCTTCTCGGT
**Mfn2**	CATCAGTTACACCGGCTCTAACT	GAGCCTCGACTTTCTTGTTCA
**Fis1**	ATGTCTTCTACCTGGCCGTG	CCATGCCTACCAGTCCATCT
**Drp1**	GCTAGATGTGCCAGTTCCAG	CATTACTGCCTTTGGGACAC
**Tnfα**	CGTCGTAGCAAACCACCAAG	GGCAGAGAGGAGGTTGACTT
**Il-6**	CCGGAGAGGAGACTTCACAG	TCCACGATTTCCCAGAGAAC
**Cxcl1**	CCTATCGCCAATGAGCTGC	TCTGAACCAAGGGAGCTTCA
**Procol1a**	CAAGAAGACATCCCTGAAGTC	AAGCACAGCACTCGCCCTCC
**Col1a**	CGAAGGCAACAGTCGCTTCA	GGTCTTGGTGGTTTTGTATTCGA
**Mmp9**	AAAACCTCCAACCTCACGGA	GTGGTGTTCGAATGGCCTTT

### Western blot analysis

Protein lysates were prepared from liver tissues supplemented with protease inhibitor cocktails (C0001, TargetMol). Equal amounts of protein (30 μg) were resolved by SDS-PAGE analysis, blotted, and probed with antibodies as described previously [6]. The antibodies used are as follows: anti-PGC-1α (NBP1-04676; Novus), anti-GAPDH (2118; Cell Signaling), anti-SIRT2 (ab67299; Abcam), anti-GFP (M048-3; MBL), anti-Flag (M185-3; MBL), anti-Lysine (9441; Cell Signaling), and horseradish peroxidase-conjugated goat anti-rabbit (sc-2004, Santa Cruz). Immunoreactive bands were detected by a chemiluminescent reaction (Lianke, Hangzhou, China).

### Quantification of NAD^+^ levels

Tissue NAD^+^ level was analyzed with a commercial NADH/NAD quantification kit (K337-100; Biovision) as described previously [51]. NAD^+^ contents were calculated according to the standard curve, and values were normalized by the total protein concentration, determined using a Bradford assay.

### 4-HNE measurement

4-HNE was measured according to the manufacturer’s instruction (MLBIO). In brief, 20 mg of liver tissue was collected and homogenized in a volume of 300 μL PBS (pH 7.4). The homogenates were then spun down at 1600 g for 10 min, and the supernatant was applied to examine 4-HNE content using a colorimetric reaction (OD 450 nm). Results were expressed as pg/mg protein, after measuring the total protein concentration with a BCA protein assay kit (Thermo Fisher Scientific). All procedures were performed at 4°C or specific temperature indicated elsewhere.

### Other biochemical analysis

Hepatic TG and TC contents, serum levels of AST and ALT were measured as described previously [52].

### Seahorse cellular flux assay

A total of ~1 × 10^5^ hepatocytes from WT and LivPGC1α mice were seeded onto a gelatin-coated culture plate 2 h before measurement using the XFe96 Seahorse intracellular flux analyzer (Agilent). Cartridges were hydrated by calibrant for 24 h before measurement in a non-CO_2_ incubator at 37°C. For mito-stress profile analysis, oligomycin (1 μg, Agilent), FCCP (2 μM, Agilent), and antimycin (1 μM, Agilent) + rotenone (1 μM, Agilent) were sequentially injected into the assay medium. The reactions measuring oxygen consumption rate (OCR) were performed in triplicate. The XFe96 Seahorse instrument was utilized following the manufacturer’s instructions:

Instrument calibration → then Mix 2 min + Measure 2 min for 3 cycles; followed by first injection (Port A); then Mix 2 min + Measure 2 min for 3 cycles; followed by second injection (Port B); then Mix 2 min + Measure 2 min for 3 cycles; followed by third injection (Port C); then Mix 2 min + Measure 2 min for 3 cycles, finish. The results were normalized by cell number in each well and analyzed via Wave software (Version 2.4, Seahorse Bioscience).

### ROS measurement

ROS levels were determined by measuring the oxidative conversion of cell-permeable 2ʹ,7ʹ-DCF diacetate (DCFH-DA, Beyotime) to fluorescent DCF (Ex 488 nm and Em 525 nm), as described previously [53]. Briefly, the cells were isolated and harvested by enzyme digestion, washed three times with PBS, and treated with serum-free culture medium (DMEM containing 10 µM DCFH-DA). After incubation for 20 min at 37°C, the cells were washed twice with PBS and the mean fluorescent intensity was measured on the LSRFortessa flow cytometer (BD Biosciences) using the FITC channel. ROS levels are expressed as a histogram of the fluorescence generated by 10,000 cells.

### Sirtuin activity

Hepatic tissues were freshly excised and snap-frozen for the measurements. Sirtuin activity was analyzed with a fluorometric sirtuin activity assay kit (K324-100; Biovision) as per the manufacturers’ instructions.

### Transmission electronic microscopy analysis

Electron microscopy was performed as described previously [54]. Briefly, liver biopsies from different diet treatments were fixed in 2.5% glutaraldehyde for 24 h at room temperature, and then postfixed in 1% OsO_4_ followed by 2% uranyl acetate. After ethanol and propylene oxide dehydration and embedding in polybed 812 resin (Polysciences, 025950-1), thin sections (80 nm) were poststained with 2% uranyl acetate followed by 0.3% lead citrate. Sample sections were viewed using a TECNAI 10 transmission electron microscope (FEI) at 80 keV.

### Statistical analysis

Statistical analyses were performed using Prism 7 (GraphPad Software Inc.) and Image J software (version 1.48). The group sizes were determined basing on previously published experiments and previous experience in which differences were observed. Shapiro-Wilk test was applied for the normality test. Unpaired Student’s *t*-test (two-tailed) was used to compare two normally distributed data sets. One-way ANOVA was used, where appropriate, to compare more than two data sets, and we chose the Tukey post hoc test when comparing every mean with every other mean; while the Sidak post hoc test was used when comparing selected pairs of means, with the selection based on experimental design. A *P* value of <.05 was considered to be statistically significant. All data are shown as mean ± standard deviation (SD).

## Supplementary Material

lnac031_suppl_Supplementary_Figure_S1

lnac031_suppl_Supplementary_Figure_S2

lnac031_suppl_Supplementary_Figure_Legend
