# Supplementary figures and images for: Peroxisome proliferator-activated receptor γ coactivator 1α maintains NAD+ bioavailability protecting against steatohepatitis
Source: Life Med. 2022 Aug 17;1(2):207–20. doi: 10.1093/lifemedi/lnac031 (PMC11749270; doi:10.1093/lifemedi/lnac031)

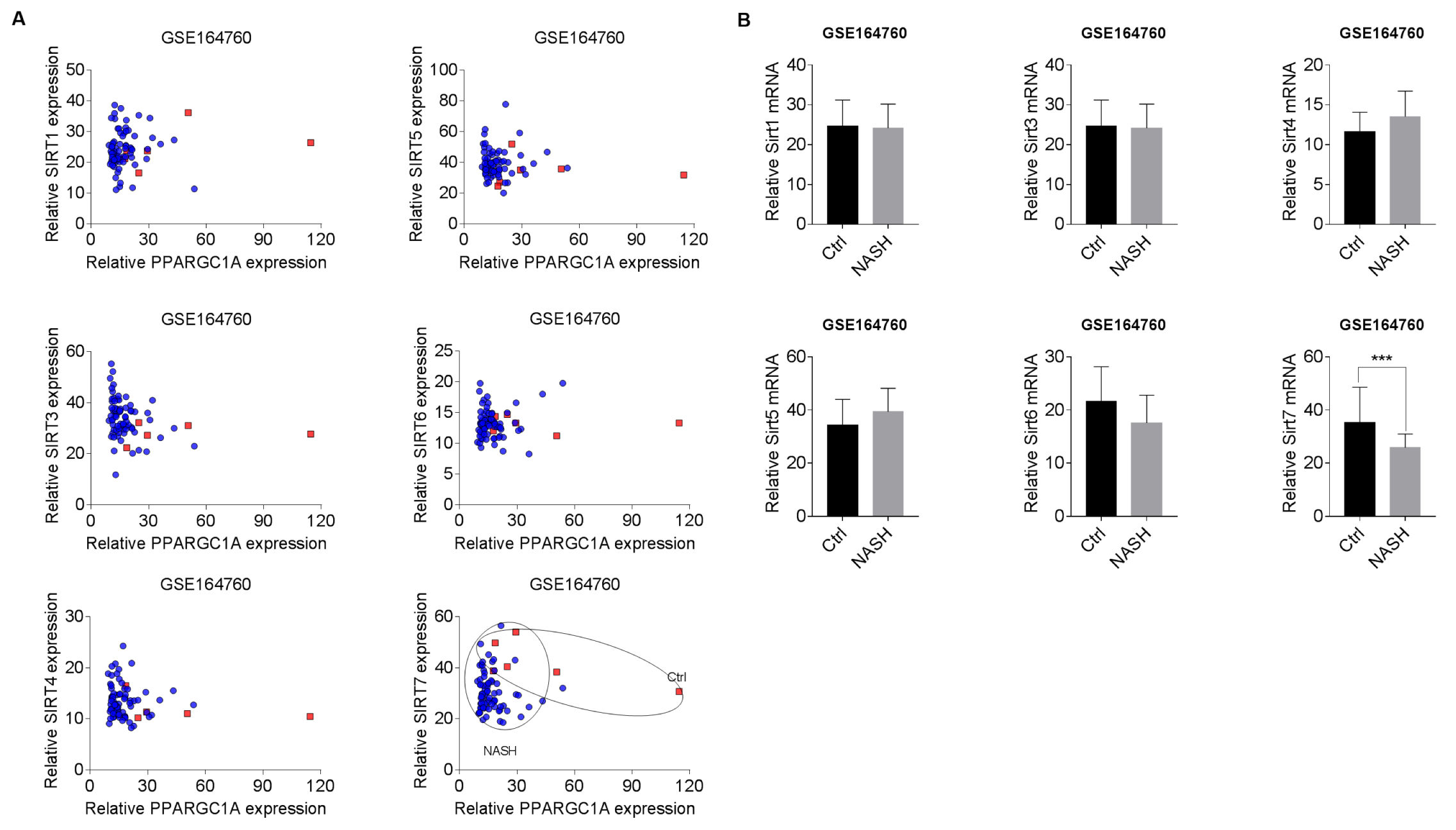

Supplement: lnac031_suppl_Supplementary_Figure_S1 [file lnac031_suppl_Supplementary_Figure_S1.png]

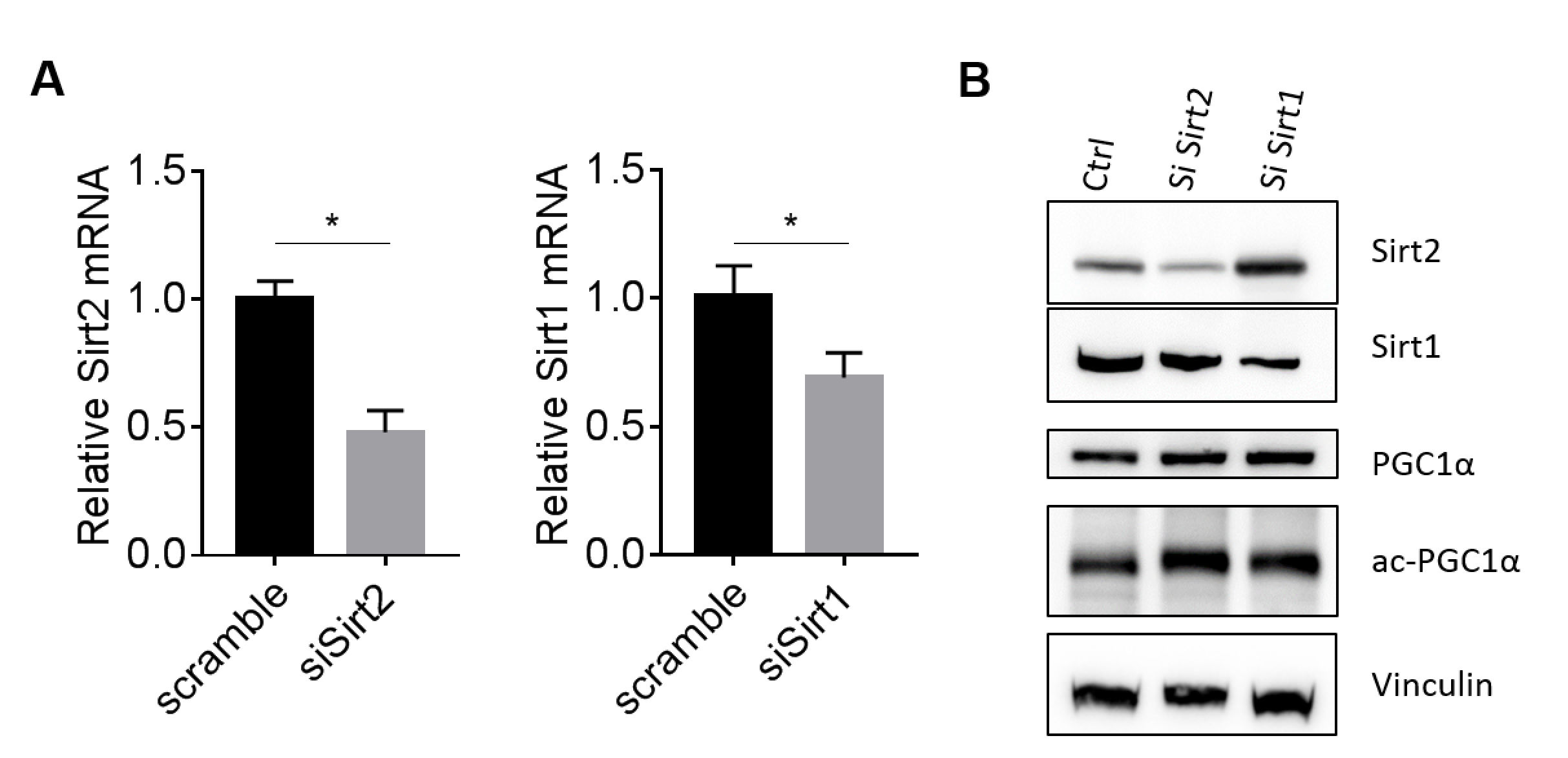

Supplement: lnac031_suppl_Supplementary_Figure_S2 [file lnac031_suppl_Supplementary_Figure_S2.png]
