# Supplementary material for: Peroxisome proliferator-activated receptor γ coactivator 1α maintains NAD+ bioavailability protecting against steatohepatitis
Source: Life Med. 2022 Aug 17;1(2):207–20. doi: 10.1093/lifemedi/lnac031 (PMC11749270; doi:10.1093/lifemedi/lnac031)
Supplement: lnac031_suppl_Supplementary_Figure_Legend [file lnac031_suppl_Supplementary_Figure_Legend.docx]

**Figure S1. Correlation analysis between PPARGC1a and SIRTs expression in human NASH biopsies.**

(A) The absence of correlation between reduced PGC-1α and down-regulated SIRTs expression in human healthy (Ctrl) and NASH biopsies, except SIRT7 (data from GSE164760). *n* = 6 (Ctrl, red square) or 74 (NASH, blue dot). (B) The relative sirtuin expression in human healthy (Ctrl) and NASH biopsies, except SIRT7 (data from GSE164760). *n* = 6 (Ctrl) or 74 (NASH). ****P* < 0.001.

**Figure S2. The acetylated PGC-1α, but not total PGC-1α, is increased upon siRNA transfection.**

(A) The relative expression of Sirt1 and Sirt2 in HepG2 cells upon the transfection of scramble siRNA or siRNA against Sirt1/2. (B) The protein expression of SIRT1, SIRT2, acetylated PGC-1α, and PGC-1α in HepG2 cells transfected with scramble siRNA or siRNA against Sirt1/2. Vinculin was used as the loading control. **P* < 0.05.
